# Adherence to dietary guidelines is associated with a lower risk of long-term cardiovascular mortality after myocardial infarction: a prospective analysis in the Alpha Omega Cohort

**DOI:** 10.1016/j.ajpc.2025.101056

**Published:** 2025-07-03

**Authors:** Esther Cruijsen, Iris van Damme, Anniek C. van Westing, Nadia E. Bonekamp, Charlotte Koopal, Frank L.J. Visseren, Johanna M. Geleijnse

**Affiliations:** a Division of Human Nutrition and Health, Wageningen University & Research, PO Box 17, Wageningen 6700 AA, the Netherlands; b Department of Vascular Medicine, University Medical Center Utrecht, Utrecht University, Utrecht, the Netherlands

**Keywords:** Diet quality, Dietary guidelines, Cardiovascular diseases, Coronary heart disease, Atherosclerosis, Mortality, Myocardial infarction, Secondary prevention

## Abstract

**Aims:**

Dietary guidelines specifically for patients with atherosclerotic cardiovascular disease (CVD) were investigated in relation to long-term mortality after myocardial infarction (MI).

**Methods:**

We included 4365 MI patients of the prospective Dutch Alpha Omega Cohort (60–80 years, 80 % male). We created the Dutch Healthy Diet-Cardiovascular Disease (DHD-CVD) index, based on the 2023 Dutch dietary guidelines for CVD patients with dietary data from a validated 203-item questionnaire. Hazard Ratios (HRs) for CVD-related and all-cause mortality across quartiles of the DHD-CVD index (ref=Q1, low diet quality) and per 1-SD increment were estimated using multivariable Cox regression models. Effect modification by health determinants was examined through stratification. Numbers needed to eat (NNE) were calculated as 1 divided by the 10-year risk reduction between extreme quartiles.

**Results:**

The mean DHD-CVD score was 88.9 ± 14.8. During a median follow-up of 14.6 years (56,037 person-years), 2869 deaths occurred, including 1112 from CVD. High vs. low diet quality was associated with a 22 % lower risk of CVD mortality (HR:0.78, 95 %CI: 0.66, 0.93), with an HR of 0.91 (95 %CI:0.86, 0.97) per 1-SD. For all-cause mortality, HRs were 0.84 (0.76, 0.94) for high vs low and 0.93 (0.90, 0.97) per 1-SD. Associations for CVD mortality were more pronounced in patients with obesity or impaired kidney function. The NNE was 13 for CVD mortality and 77 for all-cause mortality.

**Conclusion:**

Better adherence to dietary guidelines for CVD patients was associated with lower CVD and all-cause mortality risks after MI and could be an effective strategy to lower cardiovascular risk.

## Graphical abstract

Graphical abstract on adherence to dietary guidelines for patients with cardiovascular disease in relation to long-term mortality.

**Figure:**
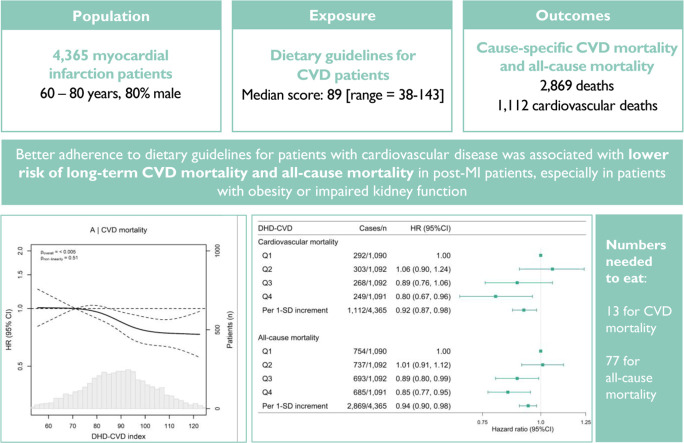

**Abbreviations**
AHEI: Alternate Healthy Eating Index
ARR: Absolute risk reduction
BMI: Body mass index
CBS: Statistics Netherlands
CHD: Coronary heart disease
CVD: Cardiovascular disease
DHD-CVD: Dutch Healthy Diet – Cardiovascular Disease
eGFR: Estimated glomerular filtration rate
FUE: Fossil energy use
FFQ: Food Frequency Questionnaire
GHGE: Green house gas emissions
HR: Hazard Ratio
Hs-CRP: high-sensitivity C-reactive protein
LU: Land use
mAHEI: modified Alternate Healthy Eating Index
MET: Metabolic equivalent of task
MI: Myocardial infarction
NHS: Nurses’ Health Study
NNE: Numbers needed to eat
NNT: Numbers needed to treat
HPFS: Health Professionals Follow-up Study
PA: Physical activity
RCS: Restricted Cubic Spline
SES: Socioeconomic status
SD: Standard deviation

## 1 Introduction

Cardiovascular diseases (CVD) are the main contributors to morbidity and mortality worldwide with an estimated number of 523 million patients living with CVD in 2019 [1]. These patients are at a high risk of a recurrent CVD event or mortality if risk factors such as elevated blood lipid levels and blood pressure are left untreated. In both the primary and secondary prevention of CVD, maintaining healthy lifestyle habits is essential, including not smoking, a healthy body weight, ample physical activity (PA) and a healthy diet [2]. A suboptimal diet is the leading modifiable risk factor for CVD globally, and it is estimated that more than one-third of CVD-related deaths could be attributed to unhealthy dietary habits [3]. Dietary guidelines, typically targeted at the general population, aim to reduce the burden of diet-related diseases including CVD and other non-communicable diseases. They serve as a standardized framework to support public education, policy making and individual health [4]. In 2023, the Dutch Health Council developed dietary guidelines specifically for atherosclerotic CVD patients [5]. These guidelines describe that the general, population-based, Dutch dietary guidelines serve as a solid basis for dietary recommendations for CVD patients [6,7], but they recommended minor adaptations. For CVD patients, it is recommended to increase fish intake to 1–2 times per week and plant sterols and stanols can be used after medical advice [5]). These guidelines are largely consistent with the dietary recommendations outlined in the 2021 European Society of Cardiology CVD prevention guideline and other international CVD guidelines [2,8,9]. In Dutch patients with established CVD, better adherence to a modified dietary index based on the general dietary guidelines was associated with a lower risk of non-fatal stroke but not with non-fatal myocardial infarction (MI) and total CVD recurrence [10]). Higher adherence to a healthy diet was associated with lower risk of CVD mortality and all-cause mortality in previous analyses of MI patients of the Alpha Omega Cohort [11]). However, follow-up time in these previous analyses was limited, and the dietary adherence score was not validated and based on outdated dietary guidelines [12]). In American and multinational CVD populations, better adherence to the dietary guidelines for Americans, reflected by the Alternate Healthy Eating Index (AHEI)-2010 and modified AHEI (mAHEI), was associated with ∼25 % lower all-cause mortality and CVD mortality risks [13,14]. The relationship between adherence to dietary guidelines and mortality risk may be modified by lifestyle and health determinants. This could have implications for risk stratification, prediction, and for tailoring dietary guidelines to specific CVD patient groups.

The objective of this study was to examine whether adherence to dietary guidelines for patients with established CVD was associated with long-term risk of CVD-related mortality and all-cause mortality in post-MI patients from the Dutch Alpha Omega Cohort. Mortality risk was also expressed as an absolute measure to enable translation and application for clinical practice. To understand how lifestyle and health determinants, including lifestyle factors, comorbidities and medication use, might modify the relationships between diet quality and mortality, exploratory subgroup analyses were performed.

## 2 Methods

### 2.1 Study design and study population

This study used prospective data from the ongoing longitudinal Alpha Omega Cohort. The cohort consists of 4837 Dutch patients aged 60–80 years who were diagnosed with an MI ≤10 years prior to enrolment. Major exclusion criteria included institutionalized living, a cancer diagnosis with short life expectancy, and moderate or severe cognitive impairment, along with several other criteria as described in full in previous publications [15,16]. In the first three years of follow-up, patients were randomly assigned to receive low doses of omega-3 fatty acids or a placebo; which did not affect major CVD events [15,16]. Baseline examinations took place between 2002–2006, and patients have been followed for cause-specific mortality since then. Written informed consent was obtained prior to study initiation. The study was approved by a central medical ethics committee and by local ethics committees at 32 participating hospitals in The Netherlands.

Patients with incomplete dietary data (*n* = 453) or implausibly high or low energy intakes (<800 or >8000 kcal/d for men, <600 or >6000 kcal/d for women; *n* = 19) were excluded, leaving a total of 4365 patients for analysis. Supplementary Figure 1 provides a flow diagram describing the selection of the population for analysis.

### 2.2 Dietary assessment

Dietary intake over the past month was assessed at baseline using a 203-item food frequency questionnaire (FFQ) which was adapted and extended from a previously validated questionnaire [17,18]. The FFQs were carefully checked by trained dieticians and missing information was collected through telephone. Intakes of energy, macronutrients and micronutrients were obtained through linkage with the Dutch Food Composition Database [19]). Salt intake was only calculated from foods since the FFQ did not include questions on discretionary salt use during food preparation or consumption.

### 2.3 Dutch healthy diet – cardiovascular disease index

Diet quality was assessed by the Dutch Healthy Diet – Cardiovascular Disease index (DHD-CVD index) using dietary intake data from the FFQ. The DHD-CVD index is a 16-component score that reflects adherence to recently published dietary guidelines for CVD patients [5]. The DHD-CVD index was based on the Dutch Healthy Diet 15 - index (DHD15-index) which is a 15-component validated score to reflect adherence to the dietary guidelines for the Dutch general population [6,12]. Compared to the original DHD15-index, the score for fish intake was modified in the DHD-CVD index to reflect higher intakes (1–2 times per week) and a component was added for use of cholesterol-lowering plant sterol or stanol-enriched products under medical supervision (any use vs. zero use). In Supplementary Table 1, an overview of the components of the DHD-CVD index and the scoring system can be found. Additionally, Supplementary Table 2 provides a list of all the food items of the FFQ included in the components. We assumed that any coffee that was consumed by this older cohort of Dutch patients was filtered.

The DHD-CVD index has a theoretical range of 0 to 160 points, with higher scores indicating better adherence to the dietary guidelines. The DHD-CVD index was categorized into quartiles (<78.98, ≥78.98 < 88.88, ≥88.88 < 98.66, ≥98.66) for studying associations with CVD and all-cause mortality. For coronary heart disease (CHD) and stroke mortality, the DHD-CVD index was categorized in tertiles (<82.44, ≥82.44 < 94.97, ≥94.97) to ensure an adequate number of cases in each category. Continuous associations of the DHD-CVD index with CVD, all-cause, CHD and stroke mortality were analyzed per 1-SD increment (14.8 points).

### 2.4 Study outcomes

CVD mortality was the primary outcome for this study. CHD mortality, stroke mortality, and all-cause mortality were secondary outcomes. Vital status was monitored through linkage with municipal registries, from baseline through December 2022. Cause-specific mortality was obtained during four follow-up phases. From 2002–2009 (Alpha Omega Trial), information was obtained from the Dutch national mortality registry (Statistics Netherlands [CBS]), treating physicians and close family members. Causes of death were coded by an independent Endpoint Adjudication Committee, as described previously [16]. After the trial through 2012, data on primary and contributing causes of death were obtained from CBS only. From 2013 through 2018, CBS provided data only on the primary cause of death and additional information on cause of death was obtained from treating physicians via a questionnaire (67 % response rate), after which coding took place by study physicians who were not involved in the current analysis. From 2018 onward, only data on primary cause of death was obtained from CBS. The endpoint CVD, CHD or stroke mortality was allocated to all patients for whom it was a primary or contributing cause of death, based on any of the data sources. During follow-up, fifteen patients were lost and were censored.

Coding was performed according to the International Classification of Diseases, Tenth Revision [20]. CVD mortality comprised codes I20-I25 (ischemic heart disease), I46 (cardiac arrest), R96 (sudden death, undefined), I15 (heart failure), and I60-I69 (stroke). CHD mortality comprised codes I20-I25, I46 and R96, stroke mortality comprised codes I60–69.

### 2.5 Covariables

Data on demographics, smoking, health status, alcohol intake and other factors were collected through a self-administered lifestyle and health questionnaire. Leisure time PA was categorized according to adherence to the Dutch PA guidelines: no PA activity, light PA (<3 metabolic equivalent of task [MET]), moderate PA (0–5 days/week of moderate or vigorous activity, ≥3 MET) and high PA (≥5 days/week of moderate or vigorous activity, ≥3 MET). Smoking status was assessed in four categories (never, former; quit >10 y ago, former; quit ≤10 y ago, current). Educational level was assessed in four categories (only elementary, low, intermediate or high). Total alcohol intake (g/d) was derived from all reported alcoholic beverages in the FFQ and classified in sex-specific categories: abstainers (0 g/d), light drinkers (>0–10 g/d for males; >0–5 g/d for females), moderate drinkers (>10–30 g/d for males; >5–15 g/d for females) and heavy drinkers (>30 g/d for males; >15 g/d for females) [21].

Body weight and height were measured by trained staff. BMI was calculated as kg/m^2^ and obesity was defined as BMI ≥30 kg/m^2^. Blood pressure was measured twice using an automatic device (HEM-711; Omron) and averaged. Hypertension was defined as use of anti-hypertensive medication or newly diagnosed based on systolic blood pressure ≥140 mmHg or diastolic blood pressure ≥ 90 mmHg. Serum lipids, plasma glucose and serum high-sensitivity C-reactive protein (hs-CRP) were determined using standard assay kits and an automated analyzer (Hitachi 921; Roche Diagnostics). Self-reported medication was coded according to the Anatomical Therapeutic Chemical Classification system [22]. Prevalent diabetes was defined as either a self-reported physician diagnosis, the use of antidiabetic medication, or elevated plasma glucose (≥126 mg/dL if fasted >4 h or ≥200 mg/dL mmol/L if nonfasted). Impaired kidney function was defined as estimated glomerular filtration rate (eGFR) <60 ml/min/1.73 m^2^, and eGFR was calculated using the 2021 CKD-EPI (Chronic Kidney Disease Epidemiology Collaboration) Jaffe creatinine-cystatin C equation [23].

Socioeconomic status (SES) was obtained using an area-based SES indicator on zip-code level from CBS [24]. This indicator includes information on the percentile of welfare, educational level and recent labor activities of the households in the area. Both negative and positive values are possible, a higher score indicates a higher SES. Self-rated health was assessed using the question: “How do you rate your overall health at this moment?”, with a 5-point answering scale ranging from poor to excellent. Environmental indicators of food products including green house gas emissions (GHGE), land use (LU) and fossil energy use (FEU) were assessed using Life Cycle Assessment [25].

### 2.6 Statistical analysis

Baseline characteristics were presented as mean ± SD for normally distributed data, median (IQR) for skewed variables and n ( %) for categorical data. Missing data were imputed using single imputation methods with bootstrapping, additive regression and predictive mean matching based on non-missing patient characteristics to minimize loss of statistical power and possible bias. Missingness was highest for the SES indicator (*n* = 634, 15 %), LDL-cholesterol (*n* = 309, 7 %), eGFR (*n* = 231, 5 %), hs-CRP (*n* = 156, 4 %), high-density lipoprotein (HDL)-cholesterol, total cholesterol and triglycerides (*n* = 111, 3 %). Missingness for other covariables was <1 %.

Multivariable Cox proportional hazards models were used to estimate hazard ratios (HRs) and 95 % confidence intervals (95 %CI) for the DHD-CVD index in quartiles and per 1-SD increment (i.e., 14.8 points) for CVD and all-cause mortality and in tertiles and per 1-SD increment for CHD and stroke mortality. A crude model was included and the lowest category was used as reference group. HRs in the first adjusted model were adjusted for age, sex and energy intake. Model 2, the main model, was additionally adjusted for SES, PA, and smoking status. Model 3 was additionally adjusted for possible intermediate factors including low-density lipoprotein (LDL)-cholesterol, systolic blood pressure and hs-CRP. We additionally estimated HRs for individual components of the DHD-CVD index per 1-SD increment adjusting for variables in model 2 and all other individual components of the DHD-CVD index. Energy adjustment was performed by including energy as covariable in model 1 according to the “standard multivariate model” as described by Willett et al. [26].

Restricted Cubic Spline (RCS) analysis was used to investigate possible non-linearity of the associations between the DHD-CVD index and mortality risk. HRs were obtained using model 2 and the median DHD-CVD index of the first quartile (71.2 points) was used as reference. The optimal number of knots was selected based on the Akaike’s information criterion (AIC) of the best fitting model and placed on the 20th, 40th, 60th and 80th percentile. The Wald chi-square statistic was used to test the non-linearity of the association.

Associations for the DHD-CVD with mortality risk were stratified for several *a priori* defined lifestyle and health determinants to investigate possible effect modification. We stratified for sex, diabetes (yes/no), obesity (yes/no), impaired kidney function (yes/no), SES (below/above a score of 0.0), self-rated health (poor or moderate/good, very good or excellent), smoking status (no or former > 10 years ago / yes or former ≤ 10 years ago) and physical activity (low/high).

In a sensitivity analysis, we excluded all patients that did not use statin therapy. Secondly, we excluded the first two years of follow-up in a sensitivity analysis to investigate possible reverse causation bias due to deteriorating health in this patient population. Lastly, we investigated associations between the DHD-CVD index and mortality risks for different phases during follow-up (trial phase, baseline through 2021, and baseline trough 2018).

Absolute risk reductions (ARR) over 10 years of follow-up of CVD and all-cause mortality were calculated based on multivariable adjusted 10-year survival probabilities for each quartile [27]. The ARR for Q4 vs Q1 was calculated as the 10-year survival probability of the quartiles relative to each other. Subsequently, the numbers needed to eat (NNE), an adapted version of the numbers needed to treat (NNT), were calculated by dividing 1 by the ARR.

Two-sided P-values of <0.05 were considered statistically significant. All statistical analyses and data visualization were performed using R version 4.0.2 (R Foundation for Statistical Computing) [28].

## 3 Results

In Table 1 the baseline characteristics of the total cohort and across quartiles of the DHD-CVD index can be found. Patients were on average 69 years old, predominantly male (79 %) and Caucasian. Most patients used lipid-modifying medication (87 %) or anti-hypertensive medication (90 %). Median time since last MI diagnosis prior to inclusion was 3.7 years. Patients with better adherence to the dietary guidelines were more often female, had higher PA levels, were more often never smokers, were less often heavy drinkers and had lower energy intakes. Their dietary patterns were more environmentally sustainable, as indicated by lower green house gas emissions, land use and fossil energy use.

**Table 1:** Baseline characteristics of 4365 patients of the Alpha Omega Cohort, overall and across quartiles of the DHD-CVD index^1^.

		Quartiles of the DHD-CVD index			
	Total population (*n* = 4365)	Quartile 1 <78.98 (*n* = 1090)	Quartile 2 ≥78.98 – 88.88 (*n* = 1092)	Quartile 3 ≥88.88 – 98.66 (*n* = 1092)	Quartile 4 ≥98.66 (*n* = 1090)
DHD-CVD index	88.8 ± 14.8	69.8 ± 7.3	84.0 ± 2.8	93.5 ± 2.7	107.7 ± 7.2
Age, y	69.0 ± 5.56	68.36 ± 5.55	68.83 ± 5.54	69.26 ± 5.54	69.55 ± 5.53
Males	3432 (78.6)	922 (84.6)	863 (79.0)	857 (78.5)	790 (72.4)
Years since last MI	3.7 [1.7, 6.3]	3.9 [1.7, 6.5]	3.8 [1.7, 6.3]	3.6 [1.5, 6.3]	3.4 [1.6, 6.2]
Physical activity^2^					
No activity	231 (5)	76 (7)	62 (6)	49 (5)	44 (4)
Light activity	1565 (36)	430 (40)	407 (37)	371 (34)	357 (33)
Moderate activity	1644 (38)	401 (37)	410 (38)	431 (40)	402 (37)
High activity	925 (21)	183 (17)	213 (20)	241 (22)	288 (26)
Smoking					
Never	723 (16)	113 (10)	173 (16)	205 (19)	232 (21)
Former, quit ≤10y ago	767 (17)	151 (13)	199 (18)	206 (19)	211 (19)
Former, quit >10y ago	2162 (49)	546 (50)	549 (50)	528 (48)	539 (49)
Current	713 (16)	280 (25)	171 (16)	153 (14)	109 (10)
Educational level					
Only elementary	889 (20)	247 (23)	240 (22)	205 (19)	197 (18)
Low	1569 (36)	398 (37)	396 (36)	402 (37)	373 (34)
Moderate	1371 (31)	332 (31)	347 (32)	336 (31)	356 (33)
High	536 (12)	113 (10)	109 (10)	149 (14)	165 (15)
High socioeconomic status^3^	2598 (60)	633 (58)	629 (58)	660 (60)	676 (62)
Alcohol^4^					
Abstainers	1336 (31)	290 (27)	350 (32)	350 (32)	346 (32)
Light drinkers	1125 (26)	211 (19)	271 (25)	299 (27)	344 (32)
Moderate drinkers	1212 (28)	304 (28)	286 (26)	305 (28)	317 (29)
Heavy drinkers	692 (16)	285 (26)	185 (17)	138 (13)	84 (8)
BMI, kg/m^2^	27.7 ± 3.8	27.9 ± 3.9	27.9 ± 4.0	27.7 ± 3.7	27.5 ± 3.7
Obesity^5^	1036 (24)	277 (25)	274 (25)	242 (22)	243 (22)
Blood lipids, mg/dL^6^					
Cholesterol	178.7 [156.2, 203.5]	181.3 [160.2, 207.4]	177.2 [155.9, 200.3]	179.5 [156.2, 204.5]	177.2 [155.1, 201.7]
LDL-cholesterol	95.9 [76.5, 116.8]	97.5 [77.3, 120.6]	94.4 [76.5, 114.1]	96.3 [76.9, 116.8]	95.1 [75.5, 116.0]
HDL-cholesterol	47.5 [40.6, 57.2]	47.9 [40.2, 57.2]	46.8 [40.2, 56.0]	47.5 [40.6, 57.2]	48.3 [41.0, 57.6]
Triglycerides	146.1 [106.3, 204.6]	149.7 [108.9, 212.6]	146.1 [107.2, 207.3]	145.2 [111.6, 199.3]	143.7 [102.7, 200.2]
Hs-CRP, mg/L	1.71 [0.81, 3.75]	1.97 [0.88, 4.13]	1.81 [0.88, 3.95]	1.63 [0.77, 3.57]	1.53 [0.74, 3.46]
Blood pressure, mmHg					
Systolic blood pressure	141.9 ± 21.6	140.9 ± 21.9	142.4 ± 21.9	142.0 ± 21.1	142.4 ± 21.4
Diastolic blood pressure	80.3 ± 11.2	80.3 ± 11.0	80.3 ± 11.7	80.2 ± 11	80.2 ± 11.0
Diabetes Mellitus^7^	883 (20)	215 (20)	247 (23)	207 (19)	214 (20)
Kidney disease^8^	692 (16)	162 (15)	160 (15)	175 (16)	195 (18)
Hypertension^9^	4161 (95)	1043 (96)	1046 (96)	1037 (95)	1035 (95)
Self-rated health					
Excellent	143 (3)	26 (2)	38 (4)	43 (4)	36 (3)
Very good	367 (8)	101 (9)	70 (6)	101 (9)	95 (9)
Good	2839 (65)	683 (63)	730 (67)	705 (65)	721 (66)
Moderate	975 (22)	266 (24)	242 (22)	235 (22)	232 (21)
Poor	41 (1)	14 (1)	12 (1)	8 (1)	7 (1)
Medication use					
Lipid modifying medication	3785 (87)	907 (83)	960 (88)	960 (88)	958 (88)
Anti-hypertension medication	3919 (90)	980 (90)	996 (91)	972 (89)	971 (89)
Energy intake, kcal/day	1873 [1549, 2226]	1988 [1620, 2393]	1892 [1587, 2251]	1854 [1531, 2161]	1786 [1491, 2089]
Environmental indicators^10^					
Green house gas emission, kg CO_2_ equivalents/day	3.3 ± 0.9	3.5 ± 1.0	3.3 ± 0.9	3.2 ± 0.9	3.2 ± 0.8
Land use, m^2^*year/day	3.6 ± 1.1	3.8 ± 1.2	3.6 ± 1.1	3.5 ± 1.0	3.3 ± 1.0
Fossil energy use, MJ/kg/day	30.2 ± 7.8	31.3 ± 8.5	30.7 ± 7.8	30.3 ± 7.6	30.2 ± 7.2

1 Values are mean ± SD for normally distributed variables, medians [IQRs] for skewed variables or *n* ( %) for categorical or discrete variables.

2 Defined as: no activity, light activity (<3 MET), moderate activity (0–5 days/week of moderate or vigorous activity, >3 MET) and high activity (≥5 days/week of moderate or vigorous activity, >3 MET).

3 Based on socioeconomic status indicator, value above 0.0.

4 Defined as: abstainers (0 g/d), light (>0–10 g/d in males and > 0–5 g/d in females), moderate (>10–30 g/d in males and >5 – 15 g/d in females) and heavy (>30 g/d in males and > 15 g/d in females.

5 Defined as BMI ≥ 30 kg/m^2^.

6 Measured in non-fasting state.

7 Defined as self-reported physician diagnosis, use of antidiabetic medication or elevated plasma glucose.

8 Based on estimated glomerular filtration rate (eGFR) of <60 ml/min/1.73 m^2^.

9 Defined based on anti-hypertensive medication use or newly diagnosed with systolic blood pressure ≥ 140 mmHg or diastolic blood pressure ≥ 90 mmHg.

10 Obtained via life cycle assessment. DHD-CVD index: Dutch Healthy Diet Cardiovascular Disease index.

### 3.1 Adherence to dietary guidelines

Adherence to individual dietary guidelines (scores) and absolute intakes (grams/day) of foods of the total cohort and across quartiles of the DHD-CVD index can be found in Supplementary Table 3. The mean DHD-CVD index score was 88.9 ± 14.8 points, with a range from 37.8 to 142.8 points. The difference between the median scores in Q1 and Q4 was 38 points. Patients showed the best adherence to guidelines for coffee, red meat, and alcohol intake (Supplementary Figure 2). Adherence was lowest for guidelines on vegetables, nuts and processed meat. The largest variation in adherence was observed for the guidelines on fruits and tea.

### 3.2 Diet quality, its components and long-term mortality risk

The median follow-up time was 14.6 years (56,037 person-years). During follow-up, a total of 2869 patients died, with 1112 (39 %) deaths due to CVD, 659 (23 %) deaths due to CHD, and 208 (7 %) deaths due to stroke as primary or contributing cause.

**Fig. 1:**
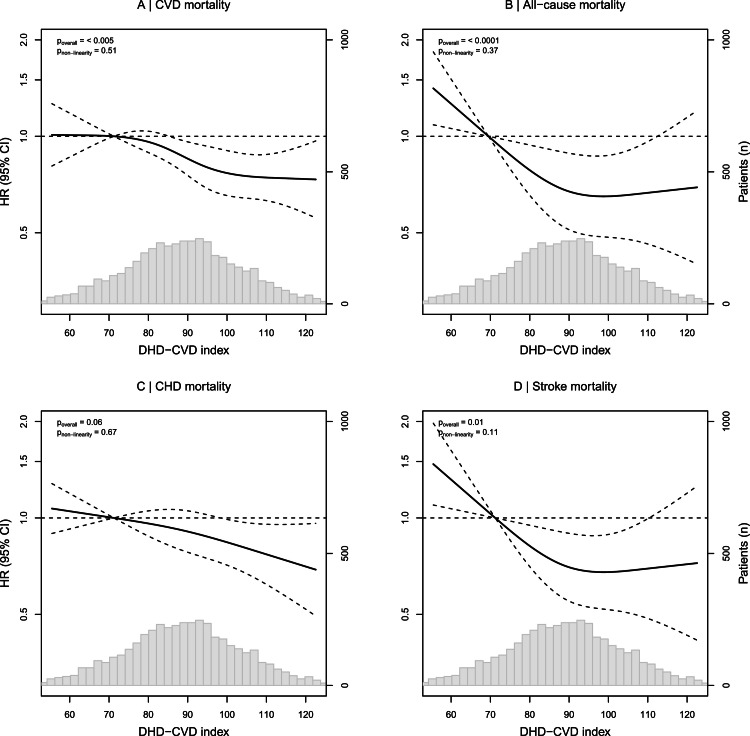
Associations for the DHD-CVD index in relation to (A) CVD mortality, (B) all-cause mortality, (C) CHD mortality and (D) stroke mortality in 4365 MI patients from the Alpha Omega Cohort. Lines are restricted cubic splines, with four knots at the 20th, 40th, 60th and 80th percentiles. The y-axis in each individual panel shows the HRs for mortality for any value of the DHD-CVD index, compared to a score of 71.2 points. Non-linearity of the association was tested with the Wald Chi-square test. DHD-CVD; Dutch Healthy Diet – Cardiovascular disease, CVD; Cardiovascular Disease; CHD; Coronary Heart Disease.

Table 2 presents HRs for CVD and all-cause mortality across quartiles and per 1-SD increment of adherence to dietary guidelines expressed as the total DHD-CVD index score. A 22 % lower risk of CVD mortality (HR: 0.78; 95 % CI: 0.66, 0.93) was observed in patients with highest (Q4) vs lowest (Q1) adherence to dietary guidelines. A 9 % lower risk of CVD mortality was observed per every 1-SD increment in adherence to the dietary guidelines (HR: 0.91, 95 % CI: 0.86, 0.97). For all-cause mortality, a 16 % lower risk was observed in patients with highest vs lowest adherence to dietary guidelines (HR: 0.84, 95 % CI: 0.76, 0.94). For every 1-SD increment in adherence, a 7 % lower risk was observed (HR: 0.93, 95 % CI: 0.90, 0.97). Higher adherence to dietary guidelines was associated with a lower mortality risk for both CHD and stroke, particularly for the latter (Supplementary Table 4). The RCS analysis showed no indication of non-linear associations (all p_non-linearity_ >0.05) for better adherence to the DHD-CVD index and risk of CVD, all-cause, CHD and stroke mortality (Fig. 1).

**Table 2:** HRs for the DHD-CVD index with CVD mortality and all-cause mortality 4365 patients from the Alpha Omega Cohort.

	DHD-CVD index				
	Quartile 1 <78.98	Quartile 2 ≥78.98 – 88.88	Quartile 3 ≥88.88 – 98.66	Quartile 4 ≥98.66	1-SD increment 14.8
n	1090	1092	1092	1091	4365
Person-years	13,665	13,751	14,163	14,458	56,037
CVD mortality					
Events	292	303	268	249	1112
Crude model	1.00	1.04 (0.88, 1.22)^1^	0.88 (0.75, 1.04)	0.80 (0.67, 0.94)	0.93 (0.88, 0.98)
Model 1^2^	1.00	0.99 (0.84, 1.17)	0.79 (0.67, 0.94)	0.68 (0.58, 0.81)	0.86 (0.81, 0.92)
Model 2^3^	1.00	1.06 (0.90, 1.24)	0.88 (0.74, 1.04)	0.78 (0.66, 0.93)	0.91 (0.86, 0.97)
Model 3^4^	1.00	1.06 (0.90, 1.24)	0.89 (0.76, 1.06)	0.80 (0.67, 0.96)	0.92 (0.87, 0.98)
All-cause mortality					
Events	754	737	693	685	2869
Crude model	1.00	0.98 (0.88, 1.08)	0.87 (0.79, 0.97)	0.84 (0.76, 0.93)	0.94 (0.91, 0.98)
Model 1	1.00	0.94 (0.85, 1.04)	0.79 (0.71, 0.88)	0.73 (0.66, 0.81)	0.88 (0.85, 0.92)
Model 2	1.00	1.01 (0.91, 1.12)	0.88 (0.79, 0.98)	0.84 (0.76, 0.94)	0.93 (0.90, 0.97)
Model 3	1.00	1.01 (0.91, 1.12)	0.89 (0.80, 0.99)	0.85 (0.77, 0.95)	0.94 (0.90, 0.98)

1 Hazard ratios (95 % CI) obtained from Cox proportional hazards models (all such values), using the lowest quartile as the reference.

2 Adjusted for age, sex and energy intake.

3 Adjusted as model 1, plus for physical activity, socioeconomic status and smoking status.

4 Adjusted as model 2, plus for LDL-cholesterol, systolic blood pressure, body mass index and hs-CRP. DHD-CVD index: Dutch Healthy Diet Cardiovascular Disease index.

Table 3 presents HRs for adherence to the individual components of the DHD-CVD index. Patients with better adherence to the guideline for nuts had a 9 % lower risk of CVD mortality (HR: 0.91, 95 % CI: 0.85, 0.97, per 1-SD increase). Additionally, a lower risk of CVD mortality was observed in patients with better adherence to the guideline for sugar sweetened beverages and fruit juices (HR: 0.92, 95 % CI: 0.87, 0.98). Adherence scores for other individual DHD-CVD index components were not significantly associated with mortality outcomes.

**Table 3:** HRs for individual components of the DHD-CVD index with CVD mortality in 4365 patients from the Alpha Omega Cohort^1^^,^^2^.

	SD	HR (95 % CI)
Per 1-SD in adherence score		
Vegetables	1.97	0.96 (0.90, 1.02)
Fruit	3.60	1.00 (0.94, 1.07)
Grain products	2.83	0.97 (0.91, 1.02)
Legumes	3.64	1.04 (0.98, 1.11)
Unsalted nuts	2.47	0.91 (0.85, 0.97)
Dairy	3.09	0.97 (0.91, 1.02)
Fish	3.68	0.99 (0.93, 1.05)
Black or green tea	4.02	0.96 (0.91, 1.03)
Unfiltered coffee	2.10	1.00 (0.95, 1.07)
Fats and oils	4.07	1.04 (0.98, 1.11)
Red meat	1.92	0.98 (0.93, 1.04)
Processed meat	3.37	1.00 (0.93, 1.07)
Sugar-sweetened beverages and fruit juices	3.59	0.92 (0.87, 0.98)
Alcohol	3.83	0.97 (0.91, 1.03)
Sodium	2.71	0.95 (0.87, 1.04)
Plant sterol or stanol-enriched products	4.92	0.96 (0.90, 1.02)

1 Classification of foods and drinks included in the DHD-CVD index is listed in Supplementary Table 2.

2 A higher score indicates better adherence to the dietary guideline for that specific component, associations were adjusted according to model 2. DHD-CVD index: Dutch Healthy Diet Cardiovascular Disease index.

### 3.3 Subgroup analyses

Supplementary Figures 3 and 4 show associations for CVD and all-cause mortality in subgroups, per 1-SD increment of the DHD-CVD index. For CVD mortality, inverse associations were slightly stronger in females (HR: 0.86, 95 % CI: 0.75, 0.97; per 1-SD increment) than in males (HR: 0.93, 95 % CI: 0.86, 0.99; per 1-SD increment) but p_interaction_ was 0.63. Also, stronger inverse associations were observed in patients with obesity (HR: 0.87, 95 % CI: 0.77, 0.98, p_interaction_ = 0.17) or impaired kidney function (HR: 0.85, 95 % CI: 0.75, 0.97, p_interaction_ = 0.03) but not in patients with diabetes (HR: 0.97, 95 % CI: 0.85, 1.10, p_interaction_ = 0.49). HRs for CVD mortality were not modified by SES and lifestyle factors. For all-cause mortality, results across subgroups were generally comparable to those for CVD mortality except for diabetes where patients with and without diabetes showed similar risks for all-cause mortality.

### 3.4 Absolute risk differences with better diet quality

For CVD mortality, an absolute 10-year risk reduction of 8 % was observed for patients with the highest adherence to dietary guidelines (Q4) compared to patients with lowest adherence (Q1). Thirteen patients would have to advance (improve their adherence to the dietary guidelines) from Q1 to Q4 to prevent one case of mortality due to CVD (10-year NNE = 13). For all-cause mortality, we observed an absolute risk reduction of 1.3 % for patients in Q4 vs Q1 (10-year NNE = 77).

### 3.5 Sensitivity analyses

After excluding the first 2 years of follow-up, the HRs did not essentially differ for CVD mortality (HR: 0.92, 95 % CI: 0.86, 0.98) and all-cause mortality (HR: 0.94, 95 % CI: 0.90, 0.97). We observed an attenuation of the associations between the DHD-CVD index and mortality with increasing follow-up time (Supplementary Table 5). The estimates for CVD mortality comparing Q4 to Q1 were HR: 0.61 at median follow-up of 3.5 years, HR:0.64 at 7.3 years, HR:0.73 at 12.4 years, and HR:0.81 at 14.6 years.

## 4 Discussion

In this prospective analysis of 4365 post-MI patients from the Alpha Omega Cohort, better adherence to dietary guidelines for CVD patients was associated with a lower risks of long-term mortality from CVD, CHD, and stroke, as well as all-cause mortality. A linear dose-response relationship was observed, with a 9 % lower risk of CVD mortality for every 1-SD increment in adherence to the DHD-CVD index. An absolute risk difference of 8 % for CVD mortality was observed between Q1 and Q4, corresponding to a NNE of 13. Associations for CVD mortality were more pronounced in patients with obesity or impaired kidney function, and weaker in patients with diabetes. Adherence to the guidelines for unsalted nuts and sugar-sweetened beverages and fruit juices, was particularly associated with a lower CVD mortality risk.

Better adherence to dietary guidelines for CVD patients was associated with lower long-term mortality risk in post-MI patients in our analyses. The associations for CVD and all-cause mortality were in line with other prospective studies in CVD patients, although they examined adherence to dietary guidelines for the general population. Analyses in 4098 MI survivors from the Nurses’ Health Study (NHS) and the Health Professionals Follow-up Study (HPFS) showed a 24 % lower all-cause mortality risk (HR: 0.76, 95 % CI: 0.60, 0.96) comparing the highest vs. lowest quintile of adherence to the 2010 Dietary Guidelines for Americans, expressed as AHEI2010 [13,29]. In this large American study, a marginally significant inverse association was observed for CVD mortality with an HR of 0.73 (95 % CI: 0.51, 1.04) that was significant in women (HR: 0.61; 95 % CI: 0.38, 0.96). The multivariable-adjusted HRs were adjusted for potential intermediate factors including diabetes, blood pressure and BMI which could have caused an attenuation of the true associations. Higher adherence to dietary guidelines, as indicated by the mAHEI score, was also associated with a lower risk of CVD mortality in 31,546 CVD patients from the multinational ONTARGET and TRANSCEND studies with risk reductions up to 35 % (HR: 0.65, 95 % CI: 0.55, 0.75) when comparing the highest vs. lowest quintile [14]. However, dietary assessment in this study was limited with only twelve food items, and the adherence score was based on whether the food item was consumed or not rather than cut-off and threshold values. Despite methodological limitations of previous studies, better adherence to dietary guidelines was consistently associated with a lower risk of mortality in CVD patients.

When we stratified our results for sex, comorbidities, indicators of health status or lifestyle factors, we observed stronger inverse associations between adherence to the dietary guidelines and mortality in patients with obesity or among those with impaired kidney function. Stronger inverse associations indicate a potential additional benefit of a healthy diet for cardiovascular risk management in these subgroups of patients. This could be attributed to less well-controlled risk factors in these patients with disease. In women, we observed a trend towards a stronger association which is in line with previous studies [13] and could also be attributed to less well-controlled risk factors in women compared to men. In patients with diabetes, adherence to dietary guidelines was inversely associated with all-cause mortality but not CVD mortality. As diabetes significantly increases cardiovascular risk [30], the potential for a healthy diet to make a significant impact on CVD mortality may be limited. Besides, a dietary pattern more tailored to manage blood glucose and other risk factors in patients with diabetes may be needed. Inverse associations for adherence to dietary guidelines with mortality risk were consistently observed independently of SES and lifestyle factors. This implies that adherence to the guidelines is important independently of these factors. There is, however, a potential uncertainty in interpreting subgroups results due to smaller sample sizes. These findings should serve as a basis for further investigations.

The NNE, based on the absolute risk reduction, shows that thirteen patients should improve their adherence to the guidelines from the lowest quartile to the highest quartile to prevent one case of CVD mortality in 10-year. This is significantly lower compared to for example statin therapy in secondary prevention, where the 5-year NNT was 83 to prevent one case of mortality, 39 for non-fatal heart attack, and 125 for stroke [31,32]. Medication use is a simple and effective strategy to lower mortality risk; however, it may also come with side effects. Improved adherence to the dietary guidelines (∼35 points from Q1 to Q4 on the DHD-CVD index) requires a more substantial change in behavior. To illustrate, this change could imply consuming 15 g of unsalted nuts daily, >100 g of legumes each week and eliminating intakes of sugar sweetened beverages. The average diet quality, measured with the DHD-CVD index, in our population is 89 out of 160 points, which is lower than the average score of 111 out of 160 points observed on a comparable index in a healthy Dutch population, indicating that an improvement of 35 points could be achieved for atherosclerotic CVD patients [33]. Behavioral change, however, is challenging due to psychological health factors and structural and social determinants [34]. Apart from changes in the living environment and food supply, this calls for novel and personalized approaches for dietary counseling [[35], [36], [37]].

A diet according to the dietary guideline consists of various nutrients that affect atherosclerotic processes. Diets high in fruits, vegetables and whole grains are linked to beneficial effects involved in the atherosclerotic process. The components of these healthy diets contribute to a more favorable lipid profile [38], reduce inflammation [39] and enhance endothelial function [40]. Management of these atherosclerotic processes is especially relevant in patients with MI to improve health and long-term outcomes. Furthermore, a low sodium content and high levels of potassium, along with other beneficial nutrients in healthy diets, are associated with reduced blood pressure levels [41]. Lowering blood pressure is key for CVD prevention, particularly stroke [42]. This may also explain stronger inverse associations for stroke mortality compared to other CVD-related mortality with better adherence to the guidelines, potentially through its effect on lowering blood pressure.

Inverse associations in our study were observed independently and alongside statin use (used by 86 % of patients), in line with previous studies on adherence to dietary guidelines and mortality risk in CVD patients [13,14]. Statin therapy is important to effectively lower LDL-cholesterol [43]. Patients of the Alpha Omega Cohort received state-of-the art statin therapy, achieving a median LDL-cholesterol level of 96 mg/dL (2.48 mmol/L), which met the treatment targets at the time of study inclusion (2002–2006) [43]. The current 2021 CVD prevention guidelines have set more strict LDL-cholesterol targets for patients with established CVD, defined as < 70 mg/mL (1.8 mmol/L) in STEP 1 and < 55 mg/dL (1.4 mmol/L) in the intensified treatment STEP 2 [2]. The observed associations in our cohort could, therefore, be less pronounced in patients currently receiving intensified LDL-treatment alongside dietary interventions. However, overall, the findings indicate the importance of an integrated approach to manage cardiovascular risk, combining pharmacological treatment with a healthy diet for optimal outcomes in CVD patients.

Strengths and limitations of this observational study should be considered. We observed stronger associations for short-term follow-up compared to long-term follow-up, which could be explained by several factors. First, dietary assessment was conducted only at baseline, without repeated measurements. This may have resulted in misclassification of patients’ adherence to the dietary guidelines, potentially leading to attenuated risk estimates for mortality. Besides, the exposure (DHD-CVD index) is closer in time to the fatal event during short-term follow-up and the baseline mortality risk increases as the cohort ages. Lastly, reverse causation bias during early follow-up may have influenced the short-term results. However, the sensitivity analyses excluding the first two years of follow-up, provided no evidence of biased risk estimates due to severe underlying disease. Salt intake, which is one of the components of the DHD-CVD index, was not accurately captured by the FFQ because the questionnaire did not cover questions on discretionary salt use. Underestimation of salt intake could have led to higher scores and stronger inverse associations. Our analyses adjusted for total energy intake to account for reporting inaccuracies from the FFQ. This adjustment, however, limits the ability to study the impact of overconsumption and excessive calorie intake, especially from energy-dense, unhealthy foods. Therefore, the impact of unhealthy eating on future mortality risk in MI patients may be underestimated in our study. Strengths of the study include the large cohort of post-MI patients in which detailed dietary data was assessed using a validated 203-item FFQ. Additionally, we had extensive data on potential effect modifiers and confounders. We studied long-term mortality risk with substantial statistical power for these endpoints. Absolute risk reductions, which are valuable for patient communication and public health, were calculated and showed that changes in dietary behaviors could be effective for lowering cardiovascular risk.

## 5 Conclusion

In conclusion, we observed lower risks of cardiovascular and all-cause mortality with better adherence to dietary guidelines specified for atherosclerotic CVD patients in Dutch post-MI patients and subgroups of these patients. Findings of this study emphasize the role of a healthy diet in reducing the risk of premature cardiovascular mortality among MI patients and support further promotion of dietary guideline implementation in clinical care.

## Data availability

Data described in the manuscript, code book and analytical code will be made available upon reasonable request pending application and approval via datasteward.hnh@wur.nl. The dataset cannot be made available due to ethical restrictions as participants did not consent to sharing their data publicly.

## CRediT authorship contribution statement

**Esther Cruijsen:** Writing – original draft, Visualization, Conceptualization, Methodology, Investigation, Formal analysis. **Iris van Damme:** Investigation, Writing – review & editing. **Anniek C. van Westing:** Writing – review & editing, Conceptualization. **Nadia E. Bonekamp:** Writing – review & editing. **Charlotte Koopal:** Writing – review & editing. **Frank L.J. Visseren:** Supervision, Writing – review & editing. **Johanna M. Geleijnse:** Writing – review & editing, Supervision, Resources.

## Declaration of competing interest

The authors declare the following financial interests/personal relationships which may be considered as potential competing interests:

Esther Cruijsen reports financial support was provided by Regio Deal Foodvalley. Iris van Damme reports financial support was provided by Regio Deal Foodvalley. C. Koopal reports financial support was provided by Regio Deal Foodvalley. Johanna M. Geleijnse reports financial support was provided by Netherlands Heart Foundation. Johanna M. Geleijnse reports financial support was provided by Regio Deal Foodvalley. Johanna M. Geleijnse reports financial support was provided by National Heart Lung and Blood Institute. Johanna M. Geleijnse reports a relationship with Jaap Schouten Foundation that includes: funding grants. Johanna M. reports a relationship with Dutch Health Council that includes: board membership. If there are other authors, they declare that they have no known competing financial interests or personal relationships that could have appeared to influence the work reported in this paper.

## Appendix Supplementary materials

Image, application 1
