# Supplementary material for: Adherence to dietary guidelines is associated with a lower risk of long-term cardiovascular mortality after myocardial infarction: a prospective analysis in the Alpha Omega Cohort
Source: Am J Prev Cardiol. 2025 Jul 3;23:101056. doi: 10.1016/j.ajpc.2025.101056 (PMC12275230; doi:10.1016/j.ajpc.2025.101056)
Supplement: Supplementary file 1 [file mmc1.pdf]

Supplementary Material

Adherence to dietary guidelines is associated with a lower risk of long-term cardiovascular mortality after myocardial infarction: a prospective analysis in the Alpha Omega Cohort

Authors: Esther Cruijsen, Iris van Damme, Anniek C van Westing, Nadia E Bonekamp, Charlotte Koopal, Frank LJ Visseren, Johanna M Geleijnse

Contents

Supplemental Figures and Tables	Page
Supplementary Figure 1 | Flow chart with selection of population for analysis.	2
Supplementary Figure 2 | Distribution and median scores of the individual DHD-CVD index components.	3
Supplementary Figure 3 | Hazard ratios for a one standard deviation increment in the DHD-CVD index in relation to CVD mortality in 4,365 MI patients from the Alpha Omega Cohort stratified for sex, diabetes, obesity, impaired kidney function, socioeconomic status, self-rated health, smoking status, physical activity, alcohol intake and statin use.	4
Supplementary Figure 4 | Hazard ratios for a one standard deviation increment in the DHD-CVD index in relation to all-cause mortality in 4,365 MI patients from the Alpha Omega Cohort stratified for sex, diabetes, obesity, impaired kidney function, socioeconomic status, self-rated health, smoking status, physical activity, alcohol intake and statin use.	5
Supplementary Table 1 | Components and Dutch dietary guidelines of the Dutch Healthy Diet Cardiovascular Disease index (DHD-CVD index) and their threshold and cut-off values.	6
Supplementary Table 2 | Classification of foods and drinks included in the DHD-CVD index in the Alpha Omega Cohort.	8
Supplementary Table 3 | Adherence to individual dietary guidelines and absolute intakes of foods and drinks in 4,365 patients of the Alpha Omega Cohort and across quartiles of the DHD-CVD index.	10
Supplementary Table 4 | HRs for the DHD-CVD index with CHD mortality and stroke mortality in 4,365 patients from the Alpha Omega Cohort	12
Supplementary Table 5 | HRs for the DHD-CVD index with CVD mortality and all-cause mortality in 4,365 patients from the Alpha Omega Cohort during different follow-up phases.	13

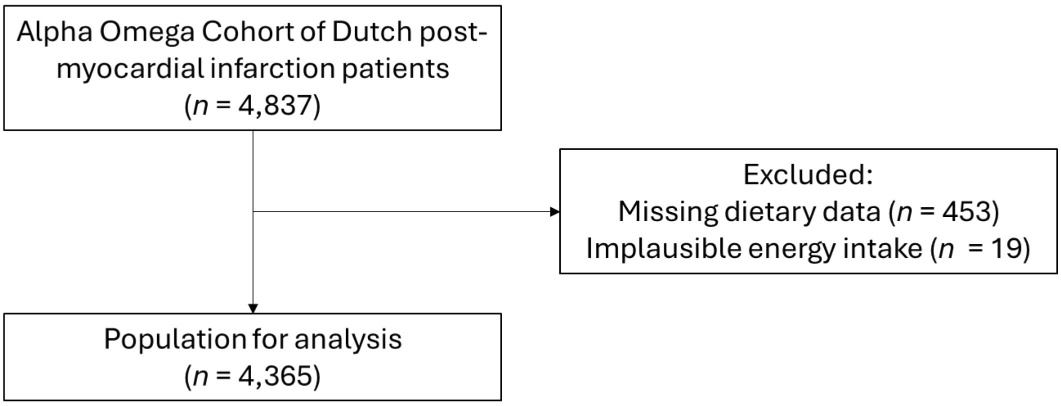

Supplementary Figure 1 | Flow chart with selection for population for analysis. Missing data for covariables was imputed. Missingness was highest for social economic status (*n* = 634), LDL-cholesterol (*n* = 309), eGFR (*n* = 231), hs-CRP (*n* = 156), HDL-cholesterol, total cholesterol and triglycerides (*n* = 111). Missingness for other covariables was <1%.

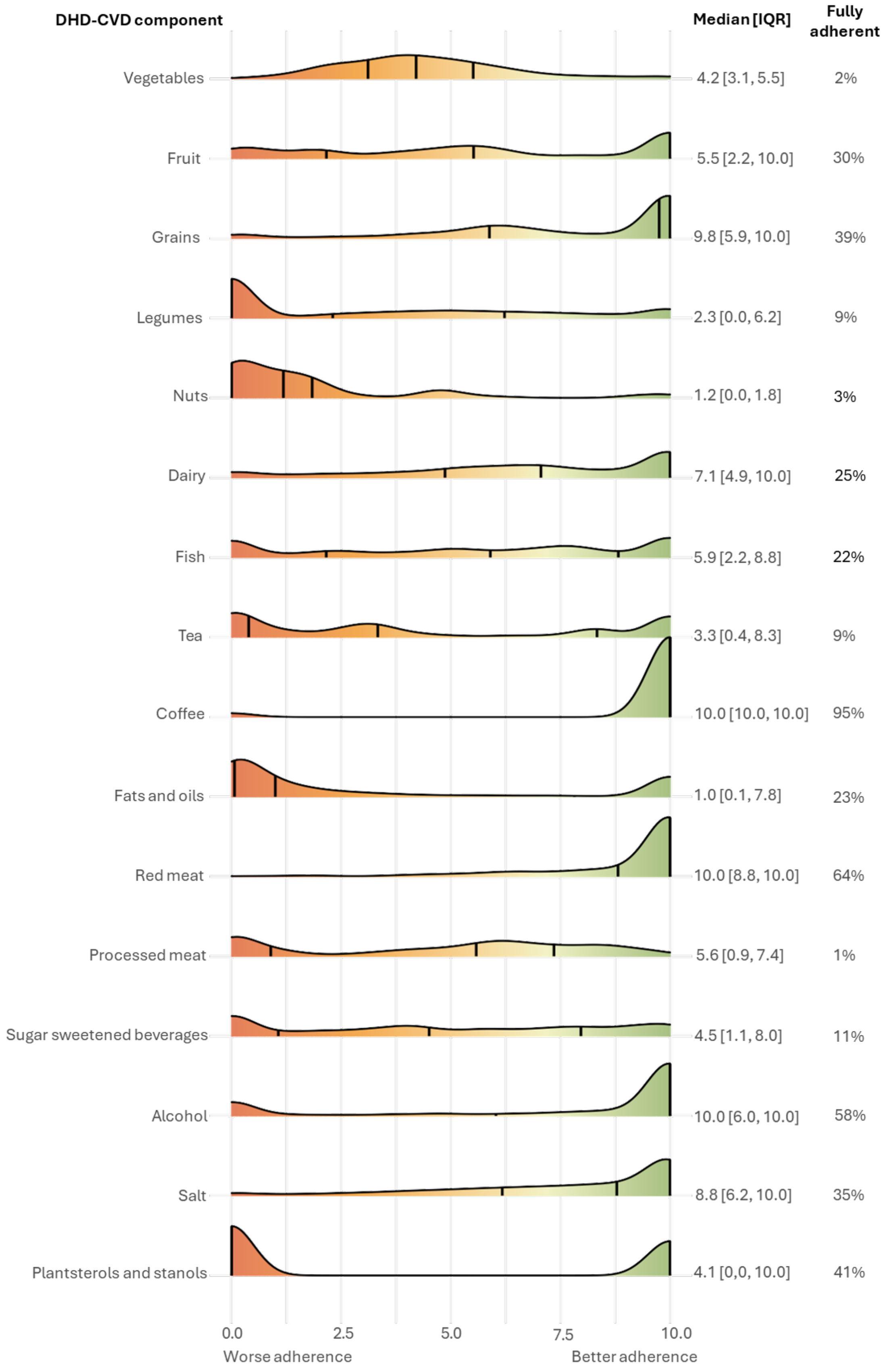

Supplementary Figure 2 | Distribution and median scores of the individual DHD-CVD index components. Fully compliant was defined as a score of 10 points for each component. DHD-CVD index; Dutch Healthy Diet Cardiovascular disease index.

CVD mortality

	Cases n / total n	HR (95% CI)	P interaction
Total population	1,112 / 4,365	0.91 (0.86, 0.97)	
Males	841 / 3,432	0.93 (0.86, 0.99)	
Females	271 / 933	0.86 (0.75, 0.97)	0.63
No diabetes	852 / 3,482	0.91 (0.84, 0.97)	
Diabetes	260 / 883	0.95 (0.84, 1.08)	0.49
No obesity	809 / 3,329	0.94 (0.87, 1.01)	
Obesity	303 / 1,036	0.86 (0.76, 0.97)	0.17
No impaired kidney function	831 / 3,673	0.93 (0.87, 1.00)	
Impaired kidney function	281 / 692	0.84 (0.74, 0.95)	0.03
Low socioeconomic status	464 / 1,767	0.90 (0.82, 0.99)	
High socioeconomic status	648 / 2,598	0.93 (0.86, 1.00)	0.69
Low or moderate self-rated health	341 / 1,016	0.93 (0.83, 1.03)	
High self-rated health	771 / 3,349	0.92 (0.86, 0.99)	0.59
No or former smoker (quit > 10 y ago)	351 / 1,490	0.87 (0.78, 0.97)	
Current or former smoker (quit < 10 y ago)	761 / 2,875	0.92 (0.85, 0.99)	0.62
Low physical activity	556 / 1,796	0.87 (0.80, 0.95)	
High physical activity	556 / 2,569	0.94 (0.87, 1.03)	0.19
Sensitivity analysis			
Patients that used statins	937 / 3,785	0.89 (0.84, 0.96)	

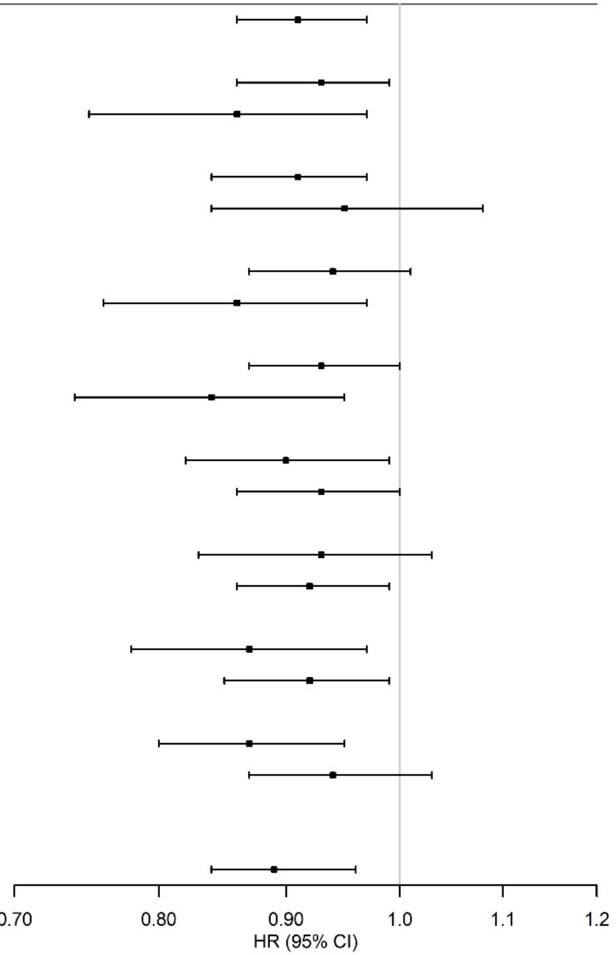

Supplementary Figure 3 | Hazard ratios for a one standard deviation increment in the DHD-CVD index in relation to CVD mortality in 4,365 MI patients from the Alpha Omega Cohort stratified for sex, diabetes, obesity, impaired kidney function, socioeconomic status, self-rated health, smoking status, physical activity, alcohol intake and statin use. Associations were adjusted according to model 2 unless variable stratified for was included.

All-cause mortality

	Cases n / total n	HR (95% CI)	P interaction
Total population	2,869 / 4,365	0.93 (0.90, 0.97)	
Males	2,236 / 3,432	0.94 (0.90, 0.98)	
Females	633 / 933	0.91 (0.84, 0.99)	0.53
No diabetes	2,211 / 3,482	0.94 (0.90, 0.98)	
Diabetes	658 / 883	0.94 (0.87, 1.02)	0.65
No obesity	2,164 / 3,329	0.95 (0.91, 0.99)	
Obesity	705 / 1,036	0.90 (0.83, 0.97)	0.24
No impaired kidney function	2,267 / 3,673	0.94 (0.90, 0.99)	
Impaired kidney function	602 / 692	0.88 (0.81, 0.96)	0.03
Low socioeconomic status	1,222 / 1,767	0.94 (0.89, 1.00)	
High socioeconomic status	1,647 / 2,598	0.93 (0.89, 0.98)	0.97
Low or moderate self-rated health	797 / 1,016	0.95 (0.88, 1.02)	
High self-rated health	2,072 / 3,349	0.94 (0.90, 0.98)	0.91
No or former smoker (quit > 10 y ago)	885 / 1,490	0.93 (0.87, 1.00)	
Current or former smoker (quit < 10 y ago)	1,984 / 2,875	0.91 (0.87, 0.96)	0.59
Low physical activity	1,326 / 1,796	0.92 (0.87, 0.97)	
High physical activity	1,543 / 2,569	0.94 (0.90, 0.99)	0.24
Sensitivity analysis			
Patients that used statins	2,148 / 3,785	0.93 (0.89, 0.97)	

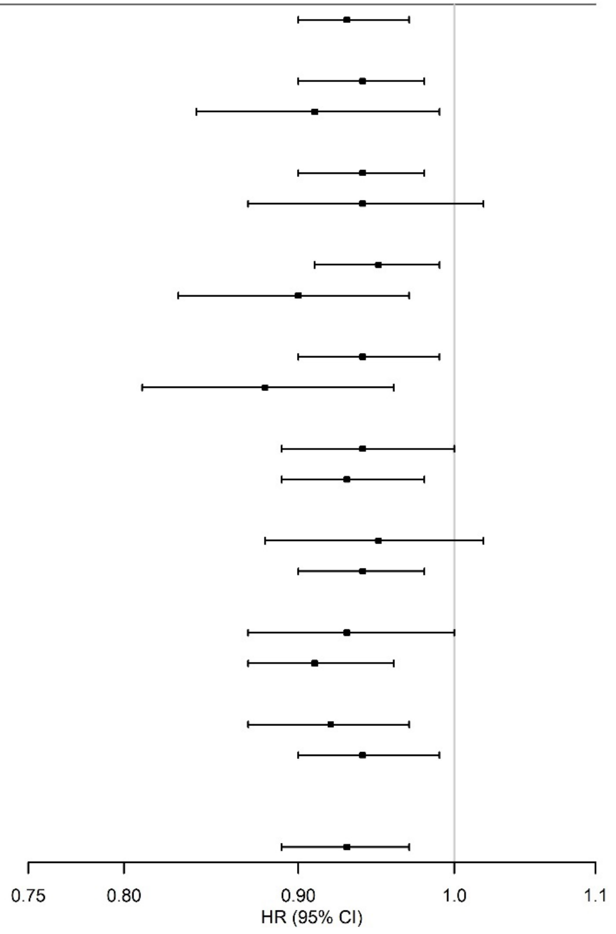

Supplementary Figure 4 | Hazard ratios for a one standard deviation increment in the DHD-CVD index in relation to all-cause mortality in 4,365 MI patients from the Alpha Omega Cohort stratified for sex, diabetes, obesity, impaired kidney function, socioeconomic status, self-rated health, smoking status, physical activity, alcohol intake and statin use. Associations were adjusted according to model 2 unless variable stratified for was included.

Supplementary Table 1 | Components and Dutch dietary guidelines of the Dutch Healthy Diet Cardiovascular Disease index (DHD-CVD index) and their threshold (minimum score) and cut-off (maximum score) values[1].

Component	Dutch dietary guidelines for CVD patients	Minimum score (=0 points)	Maximum score (=10 points)
Vegetables	Eat ≥200 g/d of vegetables	0 g/d	≥200 g/d
Fruit	Eat ≥200 g/d of fruit	0 g/d	≥200 g/d
Whole grain products[2]	a. Eat ≥90 g/d of whole grain products<br>b. Replace refined cereal products by whole grain products	No consumption of whole grain products *or* ratio of whole grains to refined ≤0.7	No consumption of refined cereal products *or* ratio of whole grains to refined grains ≥11
Legumes	Eat legumes weekly	0 g/d	≥10 g/d
Nuts	Eat ≥15 g/d of unsalted nuts	0 g/d	≥15 g/d
Dairy[3]	Eat a few portions of dairy products daily, including milk or yogurt	0 g/d OR ≥750 g/d	300-450 g/d
Fish	Eat 1-2 servings of fish (any type) weekly	0 g/d	≥21 g/d
Tea	Drink 3 cups of black or green tea daily	0 g/d	≥450 g/d
Coffee	Replace unfiltered coffee by filtered coffee[4]	0 g/d	>0 g/d
Fats and oils	Replace butter, hard margarines and cooking fats by soft margarines, liquid cooking fats and vegetable oils	No consumption of soft margarines, liquid cooking fats and vegetable oils *or* ratio of liquid cooking fats to solid cooking fats ≤0.6	No consumption of butter, hard margarines and cooking fats *or* ratio of liquid cooking fats to solid cooking fats ≥13
Red meat	Limit consumption of red meat	≥100 g/d	≤45 g/d
Processed meat	Limit consumption of processed meat	≥50 g/d	0 g/d
Sugar-sweetened beverages and fruit juices	Limit consumption of sugar-sweetened beverages and fruit juices	≥250 g/d	0 g/d

Alcohol	If alcohol is consumed at all, intake should be limited to one Dutch unit (10 g/d ethanol)	Women: ≥20 g/d ethanol<br>Men: ≥30 g/d ethanol	Women and men: ≤10 g/d ethanol
Salt	Limit consumption of table salt (sodium chloride) to 6 g/d	≥3800 mg/d sodium (9.5 g/d salt)	≤1900 mg/d sodium (4.75 g/d salt)
Plant sterol or stanol-enriched products	Consider the use of cholesterol-lowering plant sterol or stanol-enriched products	0 g/d	>0 g/d

[1] Modified from Looman et al. 2017 (1); [2] This component comprises two sub-components (a and b). Each sub-component has a maximum score of 5 points; [3] Maximum of 40 g cheese can be included; [4] For this study we assumed that all coffee that was consumed in the Alpha Omega Cohort was filtered coffee.

(1) Looman, M., Feskens, E. J., de Rijk, M., Meijboom, S., Biesbroek, S., Temme, E. H., ... & Geelen, A. (2017). Development and evaluation of the Dutch Healthy Diet index 2015. Public Health Nutrition, 20(13), 2289-2299.

Supplementary Table 2 | Classification of foods and drinks included in the DHD-CVD index in the Alpha Omega Cohort.

DHD-CVD index components	FFQ items included
Vegetables	Endive, spinach and purslane, sprouts, cauliflower, broccoli, other cabbages, carrots, peas, broad beans, green beans, leek, chicory, kohlrabi, beets, mushrooms, bell pepper, tomatoes, onion, lettuce and raw vegetables, other sorts of vegetables
Fruits	Citrus fruits, apples, pears, bananas, strawberries, blueberries, cherries, grapes, peaches, nectarines, plums, apricots, kiwis and other sorts of fruit
Whole grains[1]	Whole wheat bread, multigrain bread, whole wheat crispbread, rye bread, whole wheat rusk, fibre-rich breakfast products, whole wheat pasta, brown rice, other sorts of whole grain products
Refined grains	Crispbread, cornflakes, sweetened muesli, white bread, croissants, white rice, refined pasta, bulgur
Legumes	Capuchins, white beans in tomato sauce, brown beans, peas, white/brown beans, lentils, soybeans, green peas, chickpeas
Nuts	Nuts and seeds i.e. almonds, cashew nuts, hazelnuts, brazil nuts, peanuts, walnuts, mixed nuts, sunflower seeds,
Dairy	Luxury cheeses, reduced fat cheese, regular cheese, breakfast yogurt, yogurt, custard, milk, coffee milk, buttermilk, chocolate milk, yogurt drink, pudding, porridge, ice cream, whipped cream
Fish	Fish fingers, plaice, cod, herring, pollock, tuna, sole, anchovy, trout, pan herring, buckling, eel, sardines, salmon, mackerel, halibut
Tea	Green and black tea
Coffee	Coffee with caffeine, coffee without caffeine

DHD-CVD index components	FFQ items included
Fats and oils	Liquid cooking fats and oils<br>Different types of Halvarine products, different types of margarine products (lower in saturated fatty acids), sunflower oil, corn oil, soybean oil, safflower oil, peanut oil, olive oil, liquid frying fat,<br><br>Solid cooking fats<br>Salted and unsalted butter, semi-skimmed butter, margarines (higher in saturated fatty acids), solid frying fat, solid bake and frying fats, bacon fat
Sugar-sweetened beverages and fruit juices	Alcohol-free beer, orange juice, apple juice, grape juice, grapefruit juice, tomato juice, vegetable juice, rosehip syrup, coke with caffeine, sport drinks, breakfast drinks with fruits, milkshake with sugar, yogurt drinks with sugar, buttermilk with fruits
Unprocessed red meat	Different types of organ meat, steaks, pork, minced meat, sheep
Processed red meat	Prepared organ meat, beef, veal, pork, liver products, gammon, luncheon meat, bacon, sausage
Alcohol	Pie or cake containing alcohol, beer, low alcohol or alcohol-free beer, advocaat, mixed/long drinks, strong liquor, pudding with alcohol, pieces of chocolates with alcohol, red wine, rosé wine, white wine, sherry, vermouth, port
Sodium[2]	Cooked liver, bacon, unknown types of meat, sausage, mustard
Plant sterol or stanol-enriched products	Halvarine Becel pro.activ, margarine Benecol, Halvarine Benecol light

[1] Foods were categorized as whole grain product if they contained at least 25 % wholegrain flour.

[2] Sodium intake was only estimated from foods, because discretionary salt use could not be assessed by means of the FFQ. Sodium is present in all food items of the FFQ, except for other types of oil, solid deep frying oil, liquid deep frying oil, lard, olive oil, and deep frying oil. The food items mentioned in the table, are the foods that contribute the most to total sodium intake. Abbreviations: DHD-CVD, Dutch Healthy Diet for cardiovascular disease patients.

Supplementary Table 3 | Adherence to individual dietary guidelines (scores) and absolute intakes (grams/day) of foods and drinks in 4,365 patients of the Alpha Omega Cohort and across quartiles of the DHD-CVD index.[1]

		Quartiles of the DHD-CVD index			
	Total population (*n* = 4,365)	Q1 (*n* = 1,090)	Q2 (*n* = 1,092)	Q3 (*n* = 1,092)	Q4 (*n* = 1,091)
DHD-CVD index	88.88 [78.98, 98.66]	71.22 [65.29, 75.65]	84.07 [81.61, 86.51]	93.37 [91.25, 95.70]	106.23 [102.07, 111.64]
**Adherence to individual guidelines**					
Vegetables ≥200 g/d, score	4.2 [3.1, 5.5]	3.8 [2.6, 5.0]	4.1 [2.9, 5.3]	4.3 [3.2, 5.5]	4.8 [3.7, 6.0]
Fruit ≥200 g/d, score	5.5 [2.2, 10.0]	3.0 [0.8, 5.7]	5.2 [2.1, 9.2]	5.9 [3.4, 10.0]	9.7 [5.2, 10.0]
Grain products[2], score					
No consumption of refined cereal products *or* ratio of whole grains to refined grains ≥11	9.8 [5.9, 10. 0]	7.0 [4.8, 10.0]	9.2 [5.8, 10.0]	9.9 [6.2, 10.0]	9.9 [6.8, 10.0]
Legumes ≥10 g/d, score	2.3 [0.0, 6.2]	0.0 [0.0, 4.4]	0.0 [0.0, 5.4]	2.6 [0.0, 6.4]	5.0 [0.0, 8.2]
Unsalted nuts ≥15 g/d, score	1.2 [0.0, 1.8]	0.5 [0.0, 1.7]	0.5 [0.0, 1.8]	1.2 [0.0, 1.8]	1.2 [0.5, 4.7]
Dairy 30-450 g/d, score	7.1 [4.9, 10.0]	5.6 [2.8, 8.4]	7.1 [5.0, 9.9]	7.3 [5.3, 10.0]	8.2 [6.0, 10.0]
Fish ≥21 g/d, score	5.9 [2.2, 8.8]	3.6 [0.0, 7.1]	5.4 [1.9, 8.2]	6.7 [2.6, 9.2]	7.7 [4.4, 10.0]
Black or green tea ≥450 g/d, score	3.3 [0.40, 8.3]	1.2 [0.0, 3.3]	3.3 [0.0, 8.3]	3.3 [1.0, 10.0]	8.3 [2.8, 10.0]
Unfiltered coffee >0 g/d, score	10.0 [10.0, 10.0]	10.0 [10.0, 10.0]	10.0 [10.0, 10.0]	10.0 [10.0, 10.0]	10.0 [10.0, 10.0]
Fats and oils, score					
No consumption of butter, hard margarines and cooking fats *or* ratio of liquid cooking fats to solid cooking fats ≥13	1.0 [0.1, 7.8]	0.3 [0.0, 1.3]	0.7 [0.0, 3.6]	1.3 [0.2, 10.0]	5.7 [0.7, 10.0]
Red meat ≤45 g/d, score	10.0 [8.8, 10.0]	10.0 [7.7, 10.0]	10.0 [8.8, 10.0]	10.0 [9.1, 10.0]	10.0 [9.8, 10.0]
Processed meat 0 g/d, score	5.6 [0.9, 7.4]	3.2 [0.0, 6.3]	5.0 [0.5, 6.9]	5.7 [2.2, 7.5]	6.5 [4.9, 8.4]
Sugar-sweetened beverages and fruit juices 0 g/d, score	4.5 [1.1, 8.0]	4.0 [0.0, 7.0]	4.18 [0.6, 7.8]	4.9 [1.5, 8.3]	6.2 [2.7, 9.0]
Alcohol ≤10 g/d, score	10.0 [6.0, 10.0]	9.2 [0.0, 10.0]	10.0 [4.8, 10.0]	10.0 [8.0, 10.0]	10.0 [8.8, 10.0]
Sodium ≤1.9 g/d, score	8.8 [6.2, 10.0]	7.8 [5.0, 10.0]	8.3 [5.8, 10.0]	8.9 [6.7, 10.0]	9.4 [7.4, 10.0]
Plant sterol or stanol-enriched products, n(%) with 10 points	1,790 (41)	180 (17)	330 (30)	543 (50)	737 (68)

		Quartiles of the DHD-CVD index			
	Total population (*n* = 4,365)	Q1 (*n* = 1,090)	Q2 (*n* = 1,092)	Q3 (*n* = 1,092)	Q4 (*n* = 1,091)
**Absolute intake of DHD-CVD components**					
Vegetables, g/d	84.1 [62.2, 110.1]	76.8 [52.4, 99.9]	81.0 [57.4, 106.4]	85.2 [64.8, 110.1]	96.4 [73.2, 120.2]
Fruits, g/d	110.3 [43.2, 251.44]	60.3 [15.4, 114.5]	104.1 [41.3, 184.8]	117.4 [68.1, 268.2]	195.2 [104.8, 298.2]
Whole grains[2], g/d	116.2 [87.5, 157.92]	97.3 [70.5, 157.5]	116.7 [87.5, 158.68	119.3 [87.5, 159.6]	120.1 [87.6, 160.2]
Refined grains, g/d	10.8 [3.2, 35.0]	21.1 [3.6, 47.3]	14.1 [3.2, 36.7]	10.0 [3.2, 32.5]	7.6 [1.4, 22.3]
Legumes consumers, n(%)	2,324 (53)	443 (41)	543 (50)	590 (54)	748 (69)
Intake among consumers, g/d	6.0 [4.1, 8.7]	5.1 [3.4, 7.3]	5.4 [3.8, 8.0]	6.1 [4.3, 8.2]	6.8 [4.7, 9.5]
Nuts consumers, n(%)	2,978 (68)	643 (59)	704 (65)	790 (72)	841 (77)
Intake among consumers, g/d	1.7 [0.0, 2.7]	0.8 [0.0, 1.8]	0.8 [0.0, 2.7]	1.7 [0.0, 2.7]	1.7 [0.7, 7.0]
Dairy, g/d	271.4 [164.9, 399.0]	227.5 [127.5, 460.8]	260.9 [164.3, 401.0]	270.8 [171.0, 388.9]	30.0 [194.3, 377.6]
Fish, g/d	12.3 [4.5, 18.5]	7.6 [0.0, 15.0]	11.4 [4.0, 17.2]	14.0 [5.5, 19.4]	16.1 [9.2, 31.1]
Fatty fish, g/d	5.1 [0.9, 9.9]	3.0 [0.0, 7.1]	4.9 [0.5, 8.9]	6.0 [1.4, 10.9]	7.4 [2.6, 15.2]
Lean fish, g/d	5.5 [0.5, 11.0]	3.0 [0.0, 7.4]	5.2 [0.4, 10.3]	5.8 [1.7, 11.3]	8.1 [2.9, 14.7]
Tea, g/d	150.0 [17.5, 375.0]	54.0 [0.0, 150.0]	150.0 [0.0, 375.0]	150.0 [45.0, 450.0]	375.0 [125.0, 450.0]
Coffee, g/d	375.0 [375.0, 562.5]	375.0 [375.0, 562.5]	375.0 [375.0, 562.5]	375.0 [375.0, 562.5]	375.0 [375.0, 562.5]
Liquid fats, g/d	20.1 [12.3, 30.8]	17.6 [8.8, 28.9]	19.7 [11.3, 31.0]	20.7 [13.4, 30.9]	21.8 [14.7, 33.2]
Solid fats, g/d	10.9 [2.0, 24.2]	19.4 [9.3, 32.6]	13.1 [4.1, 26.2]	9.2 [1.4, 21.3]	2.8 [0.1, 13.1]
Red meat, g/d	36.2 [18.7, 51.5]	42.8 [21.9, 57.8]	36.5 [19.1, 51.7]	35.5 [18.7, 49.8]	28.5 [13.4, 46.1]
Processed meat, g/d	22.1 [13.2, 45.5]	34.0 [18.4, 53.1]	25.0 [15.5, 47.6]	21.3 [12.3, 38.8]	17.5 [8.2, 25.6]
Sugar-sweetened beverages and fruit juices, g/d	137.4 [50.8, 223.4]	150.0 [73.4, 295.5]	145.5 [54.0, 235.8]	127.9 [42.0, 212.5]	95.4 [25.2, 181.4]
Alcohol, g/d	7.0 [1.3, 17.4]	11.2 [1.6, 29.9]	7.0 [1.1, 20.1]	5.9 [1.1, 13.7]	5.7 [1.3, 12.3]
Sodium, mg/d	2,130.8 [1,731.6, 2,627.2]	2,317.6 [1,796.8, 2,857.1]	2,216.6 [1,753.9, 2,699.3]	2,092.9 [1,710.2, 2,514.5]	2,012.7 [1,680.8, 2,387.5]
Plant sterols/stanol product consumers, n (%)	1,790 (41)	180 (17)	330 (30)	543 (50)	737 (68)

[1] Values are means ± SDs for normally distributed variables, medians [IQRs] for skewed variables, or n (%) for categorical variables. [2] Whole grains also partly included refined grain products, such as brown bread and multigrain bread. DHD-CVD index: Dutch Healthy Diet Cardiovascular Disease index.

Supplementary Table 4 | HRs for the DHD-CVD index with CHD mortality and stroke mortality 4,365 patients from the Alpha Omega Cohort

	DHD-CVD index			
	Tertile 1	Tertile 2	Tertile 3	1-SD increment
	<82.44	≥82.44 – 94.97	≥94.97	14.8
n	1,455	1,455	1,455	4,365
Person-years	18,172	18,719	19,146	56,037
**CHD mortality**				
Events	246	215	198	659
Crude model	1.00	0.85 (0.70, 1.02)[1]	0.76 (0.63, 0.91)	0.92 (0.85, 1.00)
Model 1[2]	1.00	0.80 (0.66, 0.96)	0.67 (0.55, 0.81)	0.87 (0.80, 0.94)
Model 2[3]	1.00	0.87 (0.72, 1.04)	0.76 (0.62, 0.92)	0.92 (0.85, 1.00)
Model 3[4]	1.00	0.88 (0.73, 1.06)	0.78 (0.64, 0.94)	0.93 (0.86, 1.01)
**Stroke mortality**				
Events	82	61	65	208
Crude model	1.00	0.72 (0.52, 1.00)	0.74 (0.54, 1.03)	0.90 (0.78, 1.03)
Model 1	1.00	0.68 (0.49, 0.95)	0.65 (0.46, 0.90)	0.83 (0.73, 0.96)
Model 2	1.00	0.72 (0.52, 1.01)	0.70 (0.50, 0.97)	0.87 (0.75, 1.00)
Model 3	1.00	0.72 (0.51, 1.00)	0.69 (0.49, 0.97)	0.87 (0.75, 1.00)

[1]Hazard ratios (95% CI) obtained from Cox proportional hazards models (all such values), using the lowest tertile as the reference; [2]Adjusted for age, sex and energy intake; [3]Adjusted as model 1, plus for physical activity, socioeconomic status and smoking status; [4]Adjusted as model 2, plus for LDL-cholesterol, systolic blood pressure, body mass index and hs-CRP. DHD-CVD index: Dutch Healthy Diet Cardiovascular Disease index.

Supplementary Table 5 | HRs for the DHD-CVD index with CVD mortality and all-cause mortality 4,365 patients from the Alpha Omega Cohort during different follow-up phases.

			DHD-CVD index			
	Total cases (n)	Median follow-up time (y)	Quartile 1 <78.98	Quartile 2 ≥78.98 – 88.88	Quartile 3 ≥88.88 – 98.66	Quartile 4 ≥98.66
**CVD mortality**						
Trial phase (baseline – 3y follow-up)	140	3.5	1.00	1.09 (0.71, 1.67)[1]	0.86 (0.54, 1.37)	0.61 (0.36, 1.05)
Baseline through 31-12-2012	412	7.3	1.00	1.03 (0.79, 1.33)	0.85 (0.65, 1.12)	0.64 (0.47, 0.86)
Baseline through 31-12-2018	903	12.4	1.00	1.01 (0.84, 1.20)	0.87 (0.72, 1.05)	0.73 (0.60, 0.89)
Baseline through 31-12-2022	1,112	14.6	1.00	1.05 (0.89, 1.24)	0.89 (0.75, 1.06)	0.81 (0.68, 0.96)
**All-cause mortality**						
Trial phase (baseline – 3y follow-up)	309	3.5	1.00	1.01 (0.75, 0.36)	0.90 (0.65, 1.23)	0.81 (0.57, 1.13)
Baseline through 31-12-2012	944	7.3	1.00	0.99 (0.84, 1.18)	0.91 (0.76, 1.09)	0.82 (0.68, 0.99)
Baseline through 31-12-2018	2035	12.4	1.00	1.00 (0.89, 1.13)	0.95 (0.84, 1.08)	0.86 (0.76, 0.98)
Baseline through 31-12-2022	2,869	14.6	1.00	1.01 (0.91, 1.12)	0.89 (0.80, 0.99)	0.86 (0.77, 0.96)

[1]Hazard ratios (95% CI) obtained from Cox proportional hazards models (all such values), using the lowest quartile as the reference, and using model 2. DHD-CVD index: Dutch Healthy Diet Cardiovascular Disease index.
